# Shedding light on the environmental impact of the decomposition of perovskite solar cell

**DOI:** 10.1038/s41598-023-44781-5

**Published:** 2023-10-21

**Authors:** Negin Sabahi, Hashem Shahroosvand

**Affiliations:** Group for Molecular Engineering of Advanced Functional Materials (GMA), Chemistry Department, University of Zanjan, Zanjan, Iran

**Keywords:** Environmental sciences, Solar cells

## Abstract

Perovskite materials, as the heart of perovskite solar cells (PSC), attracted great interest in the photovoltaic community since the efficiency of PSC dramatically increased to over 25% in a short period. However, the presence of Pb metal in the perovskite crystalline limits the progress of this new generation of solar cells from environmental aspects. Here, we have systematically investigated the impact of the decomposition of perovskite material on the special plant, named Coleus. The influence of the decomposition of a perovskite solar cell (**p-PbI**_**2**_) has a three-fold lower destruction than commercial PbI_2_ (**s-PbI**_**2**_) in the same condition. The **p-PbI**_**2**_ made destroying the roots and leafs slower and smoother than **s-PbI**_**2**_, which the amount of water absorption with the plant’s root from **p-PbI**_**2**_ is two-fold lower than **s-PbI**_**2**_. The atomic absorption spectroscopy (AAS) indicated that the amount of Pb in the first week is about 3.2 and 2.1 ppm for **s-PbI**_**2**_, and **p-PbI**_**2**_, respectively, which in following for two next weeks reached to about relatively close together and finally in the last week decreased to 1.8 ppm for **s-PbI**_**2**_ and increased to 2.4 ppm for **p-PbI**_**2**_. This paper opens new avenues and challenges about the actual scenario on the impact of perovskite materials in PSCs on the plant and live metabolisms.

## Introduction

In general, perovskites are a group of structurally similar materials that are quickly synthesized and deposited on any substrate as well are excellent candidates for use in low-cost and efficient PSCs^1,2^. Superconductivity and magnetoresistance^3,4^ are unique properties that create novel applications beyond just photovoltaic devices, light emitters in PeLEDs^5^, lasers^6^, and sensing applications in humidity, temperature, gas, and solvent sensors^7–10^. Therefore, the exponentially growing of perovskite materials in many applications mentioned above, attracted great interest in the importance of studying their effects on the environment and living organisms as a key factor to commercialization of them^11^. Currently, due to the most records of the power conversion efficiency (PCE) of the PSCs based on Pb halide perovskite systems, nearly 26%, use of lead in perovskite solar cells has opened new issues about its toxicity in large- scale and marketplace. This problem shows the most important challenge because the PSC based on none-Pb perovskite indicated a poor PCE below 15%^12^.

Moreover, based on European Commission Regulation (EC) No 1881/2006, set on 19 December 2006 to set maximum levels for specific contaminants in food, last revised on 26/03/2023, the amount of lead allowed in food must range from 0.02 to 1 (ppm weight), which made use of PbI_2_ are banned in many countries^13^.

In this framework, a PSC made of a layer with a thickness of 0.6 µm of lead methylammonium iodide perovskite contains 0.8 g of lead per square meter, meaning that the percentage of lead in the perovskite structure is negligible^11^. However, due to the large surface area required for energy supply and various applications, it needs more attention. In fact, an increase in the lead concentration in the soil has a significant harmful on the growth of plants^14–16^, the body of animals and humans through the entering into food chain.

To the monitoring the trace of Pb in different media, some usual tolls have been successfully carried out. Instant, Infrared Spectroscopy confirms the toxicity of perovskite solution dispersed in a mouse cell culture medium. Benmessaoud et al. reported that perovskite MAPbI_3_ activates apoptotic cell death by damaging the plasma membrane and activating mitochondrial/intrinsic pathways^17^. In the following, A. Babayigit et al. showed that any detectable amount of lead in the blood causes severe acute and chronic damage to all organs and tissues of the body, especially the nervous system of children, which leads to lifelong mental disorders and harms human health^18^.

Although a large number of studies have been reported to minimize the conversion of CH_3_NH_3_PbI_3_ to PbI_2_^19–23^, because perovskite is an ionic crystal, intense light, moisture, and heat significantly rapid this death pathway^24^. In addition, factors such as fire, heavy rain, and shock effectively destroy any solar cell^25^, which must be prevented by various chemical and physical methods.

To address these issues, self-healing polymer capsules^21^, including chemical methods and high-melting-point materials^26^, are among the most efficient physical techniques for reducing lead leakage from deconstructed perovskite solar cells containing Pb atoms.

In addition to the key role of external factors on the destroying PSC, many reports highlighted the intrinsic and internal destruction of perovskites, which generally occurs from paths 1 and 2^27^. The most important compound that remains in any degradation process of perovskite is PbI_2_ which increases the lead concentration in the soil^28^.

1 $${\text{CH}}_{{3}} {\text{NH}}_{{3}} {\text{PbI}}_{{3}} \left( {\text{s}} \right) \to {\text{NH}}_{{2}} {\text{CH}}_{{3}} \left( {\text{g}} \right) + {\text{HI}}\left( {\text{g}} \right) + {\text{PbI}}_{{2}} \left( {\text{s}} \right),$$

2 $${\text{CH}}_{{3}} {\text{NH}}_{{3}} {\text{PbI}}_{{3}} \left( {\text{s}} \right) \to {\text{NH}}_{{3}} \left( {\text{g}} \right) + {\text{CH}}_{{3}} {\text{I}}\left( {\text{g}} \right) + {\text{PbI}}_{{2}} \left( {\text{s}} \right).$$

Therefore, it is crucial to develop and extend our knowledge about the mechanism of destruction of PSC and switch to more scientific studies on the influence of destroying the PSC and producing Pb metal on earth and living organisms.

In a brilliant paper, Junming Li et al. investigated the difference between Pb produced from perovskite and PbI_2_ contamination, simulated wasted soil by increasing different amounts in natural soil and using atomic absorption spectroscopy (AAS) to estimate the amount of Pb over 20 days and proposed that the amount of lead in leaves stems, and roots of mint plants (Mentha spicata), Capsicum annum (chili), and Brassica campestris (cabbage) grown in soil containing perovskite is significantly more than the amount of lead from plants grown in soils containing PbI_2_.

In following, regarding the influence of water contamination and not soil contamination, here we used the Coleus plant treated in contaminated water to assess the environmental impact of lead from halide perovskite on roots and roofs. To better observe all parts of the plant, we kept the plant in water and monitored the changes of all part of plant during the growth process for four weeks.

## Experimental

### Materials and equipment

**Figure 1:**
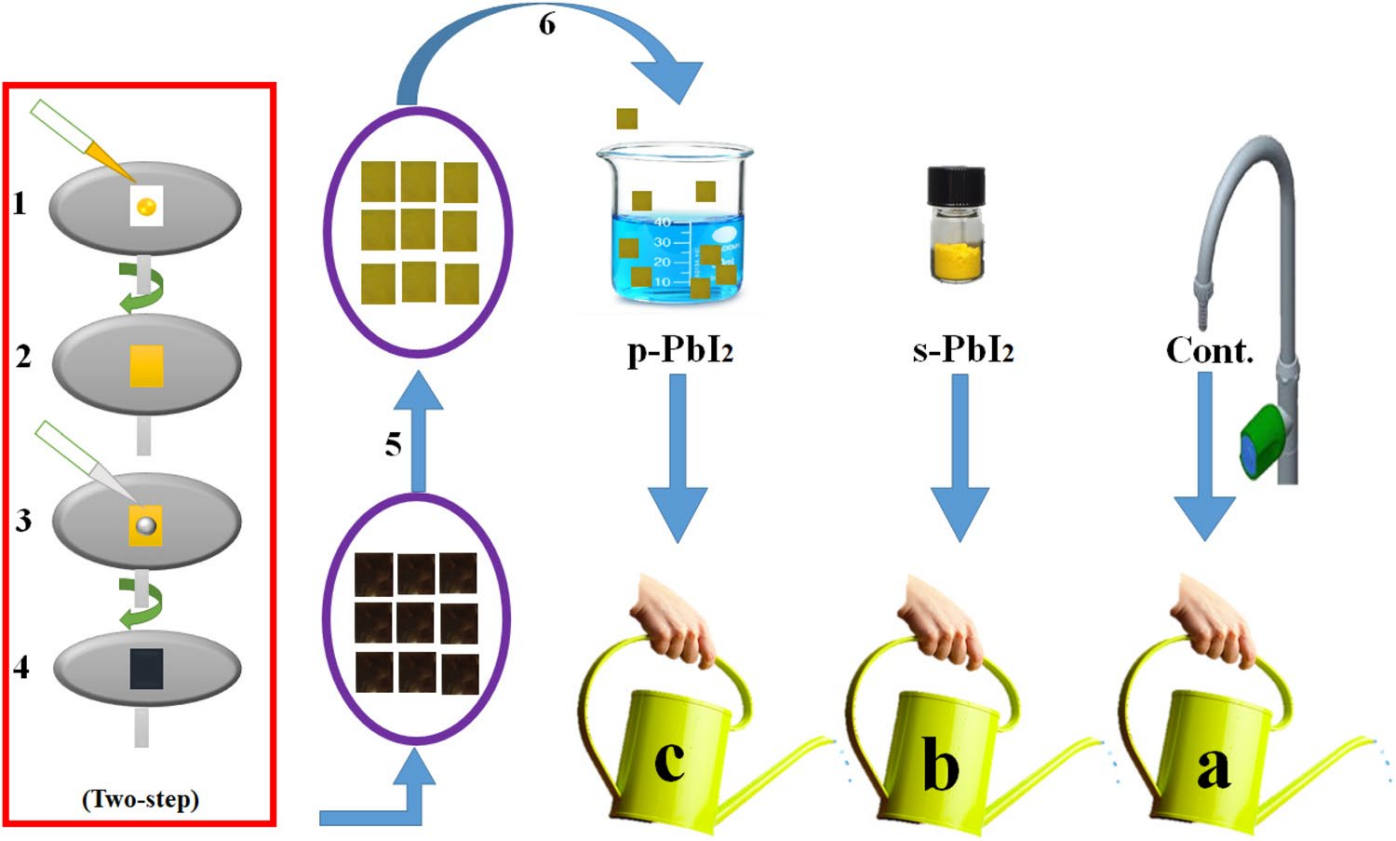
Schematic illustration of the preparation of irrigation regime with (a) drinking water, (b) **s-PbI**_**2**_, and (c) **p-PbI**_**2**_ Prepared in steps: (1) PbI_2_/DMF dripping, (2) annealing (100 °C), (3) MAI/IPA dripping, (4) Annealing (100 °C), (5) staying for two weeks, (6) dissolving in distilled water.

Plant material (Fig. 1a) was collected from the region of Gavazang located at Zanjan, IRAN (36° 39′ 51.84″ N, 48° 29′ 8.16″ E). Plant studies and collection of plant material, complied with relevant institutional, national, and international guidelines and legislation.

All materials are provided by Merck and Sigma Aldrich with a purity of 99%, and Atomic absorption spectroscopy was registered in an Analytikjena Model: novAA 350 Made in Germany.

### Experimental steps

**Figure 2:**
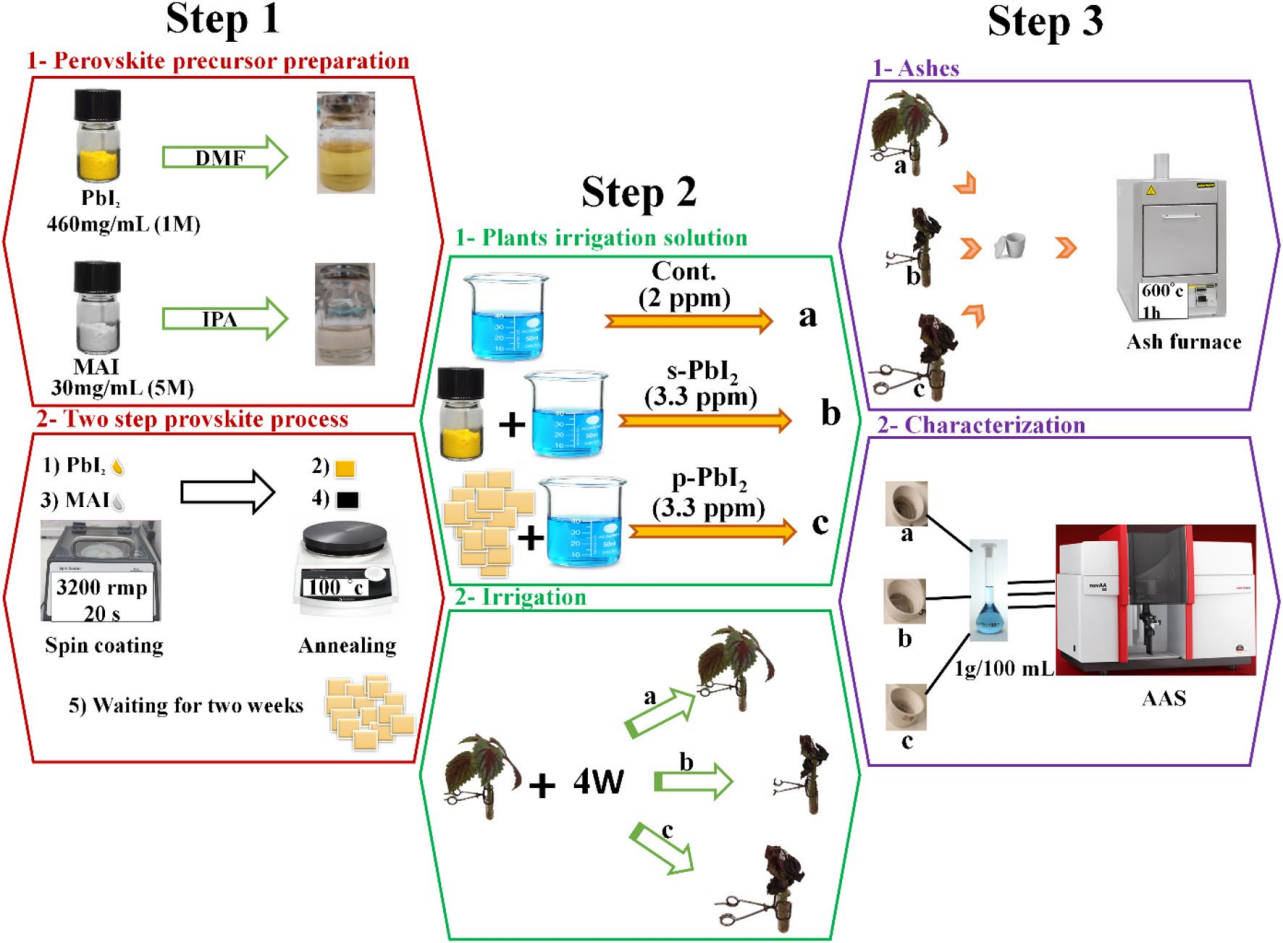
Schematic illustration of the experimental steps: Step 1: 1- perovskite precursor preparation, and 2- two-step perovskite process. Step 2: 1- plants irrigation solution, and 2-irrigation of plants. Step 3: 1- heating of plants, and 2- characterization method.

The procedure of preparation is constructed of three step which described in following:

*Step 1*: The synthesis of perovskites: first, 460 mg of PbI_2_ was mixed in 1mL dimethylformamide (DMF) and spin-coated on glass in the glove box, then 30 mg MAI was disolved in 1mL of 2-propanol (IPA) and spin-coated on prepared PbI_2_ film and placed on a hotplate at 100 °C. The color of the deposited layer went from yellow to dark brown, which indicated the formation of perovskite film (Fig. 2, step 1).

*Step 2*: The plant treatments: the plant treatments have been done in two rounds: round 1: plants irrigation solution and round 2: irrigation process as shown in Fig. 2, step 2.

To investigate the influence of PbI_2_ from decomposed perovskite and synthetic ones on the Coleus plant, the preparation of samples is explained as following, which schematically described in Fig. 1. (a) The reference sample dissolved drinking water_,_ which named **Cont.**, (b). For the preparation of the solution of synthetic PbI_2,_ which named **s-PbI**_**2**_, 7.3 mg of lead iodide was dissolved in 1 L of distilled water to give a solution of 3.3 ppm of lead, based on AAS measurements, and (c) to prepare the PbI_2_ from decomposed perovskite, namely **p-PbI**_**2**_, perovskite film was formed two-step method, as described above. After staying the films of produced perovskite in the atmosphere for two weeks without encapsulating, when dark-brown perovskite completely turned to yellow perovskite, 9.9 mg of produced perovskite dissolved in 1 L of distilled water to give a solution of 3. 3 ppm of lead. For **s-PbI**_**2**_ and **p-PbL**_**2**_ the amount of Pb was set at 3.3 ppm which confirmed by AAS.

Three samples including reference, **s-PbI**_**2**_, and **p-PbI**_**2**_ with lead concentrations of 2, 3.3, and 3.3 ppm, respectively, were added to tubes containing the Coleus plants for four weeks.

*Step 3*: the preparation of samples for AAS. After left 4 weeks, ashes of samples were prepared and 1 g of Coleus plant ash was dissolved in 100 mL distilled water, and the amount of Pb was estimated by the **AAS** method with the preparation of calibration curves (Fig. 2, step 3).

## Results and discussion

Based on the mints grown in the natural soil as a reference sample, the initial amount of Pb calculated about 12.0, 3.7, and 8.0 ppm for roots, stems, and leaves, respectively. Moreover, the amount of Pb in **p-PbI**_**2**_, especially in the stems and leaves, which are about two-fold. In brief, Junming Li et al. clearly showed that the destroying of perovskite produced a large amount of Pb compared to **s-PbI**_**2**_ ones.

**Figure 3:**
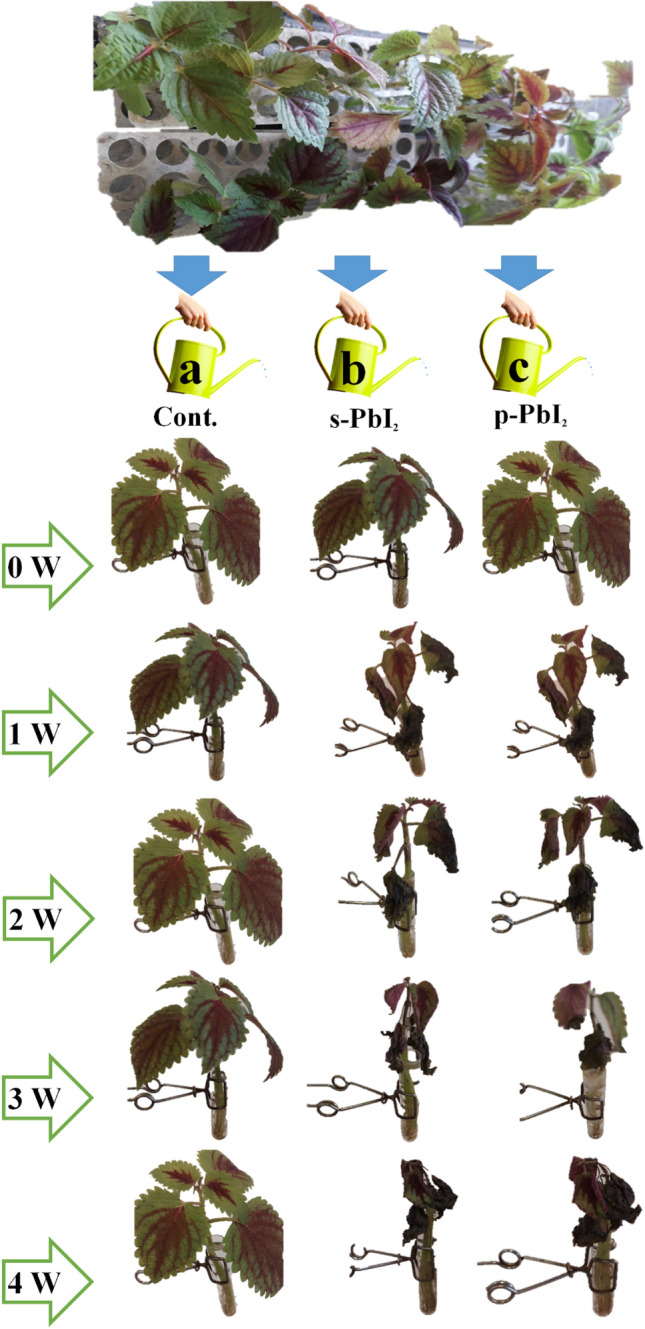
Overview of the experiment of Coleus growth in (a) irrigation water containing an initial concentration of lead of 2 ppm, (b) the concentration of lead is increased to 3.3ppm by increasing lead iodide, (c) the concentration of lead has been reduced by dissolving the yellow perovskite to 3.3 ppm.

To illustrate what happens in the roots, the Clouse plant was treated by monitoring the roots in water and carefully studied different irrigation conditions containing perovskite and fresh PbI_2_. In this research, several plant’s roots in drinking water were selected and classified into three groups and the changes were monitored for four weeks. The results as shown in Fig. 3 indicated that in series **Cont.**, the sample control group, does not change from the first to the fourth week, while in series **s-PbI**_**2**_**,** and **p-PbI**_**2**_ rinse the amount of lead, changing the color (green to brown), and becoming to smaller after 4 weeks. The leaves of series **s-PbI**_**2**_ started to collapse in the second week, while the leaves of series **p-PbI**_**2**_ began to defoliation in the third week, which finally both two samples containing PbI_2_ were destroyed after four weeks.

Some studies have been done to get deeper insight into the mechanism of interaction of Pb in plants and color changing process. Instant, N. Phuong and D.C. Valeriano reported that the multifunction interaction between anthocyanin and chlorophyll changed the color from red to green^16^. In another exciting report, Farouk S Nas and Muhammad Ali confirmed that lead metal significantly limited the formation of chlorophyll by causing reduced uptake of elements such as Magnesium and Iron in plants.

Comparison  of stability of samples treated with **s-PbI**_**2**_, and **p-PbI**_**2**_, the sample containing **s-PbI**_**2**_ was earlier decomposed than samples **p-PbI**_**2**_, while, the reference sample without any Pb source was unchanged in this period. From these observations, it concluded that the sample containing perovskite (**p-PbI**_**2**_) showed more stability than the commercial Pb (**s-PbI**_**2**_**)** sample over the same time.

**Figure 4:**
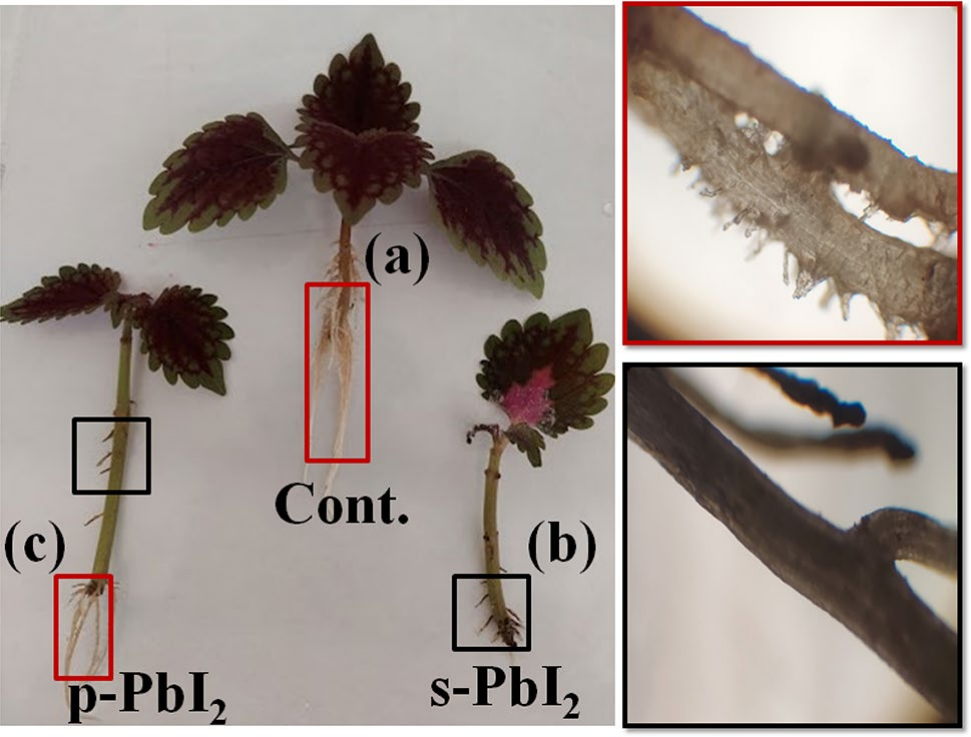
Comparison of plant’s root of plant in **Cont.**, **s-PbI**_**2**_**,** and **p-PbI**_**2**_series samples.

To investigate the effect of destroyed perovskite and PbI_2_ on the Coleus’s roots, all three samples of series **Cont.**, **s-PbI**_**2**_, and **p-PbI**_**2**_ were considered, as shown in Fig. 4, as follow: in series **Cont.**, the sample control group long, white, and clear roots after ten days, while the Coleus’s roots of series **s-PbI**_**2**_, which irrigate with PbI_2_ and excess water shown shortening and going to black color. Surprisingly, the **p-PbI**_**2**_-series plant roots, irrigated with excess water, produced both shapes of roots.

To find the mechanism of interaction of Pb with the plant’s root, in a critical report, R. Riyazuddin et al. found that in the presence of heavy metals such as pb^2+^ in samples, the amount of hydrogen peroxide as an oxidative agent was dramatically increased and consequently the roots have been completely decomposed^29^, which was earlier confirmed by Anastasia Giannakoula et al.^30^.

Given that only water-soluble substances can be absorbed by the plant, subsequent measurement of the amount of absorbed water indicated the viability of a plant.

**Figure 5:**
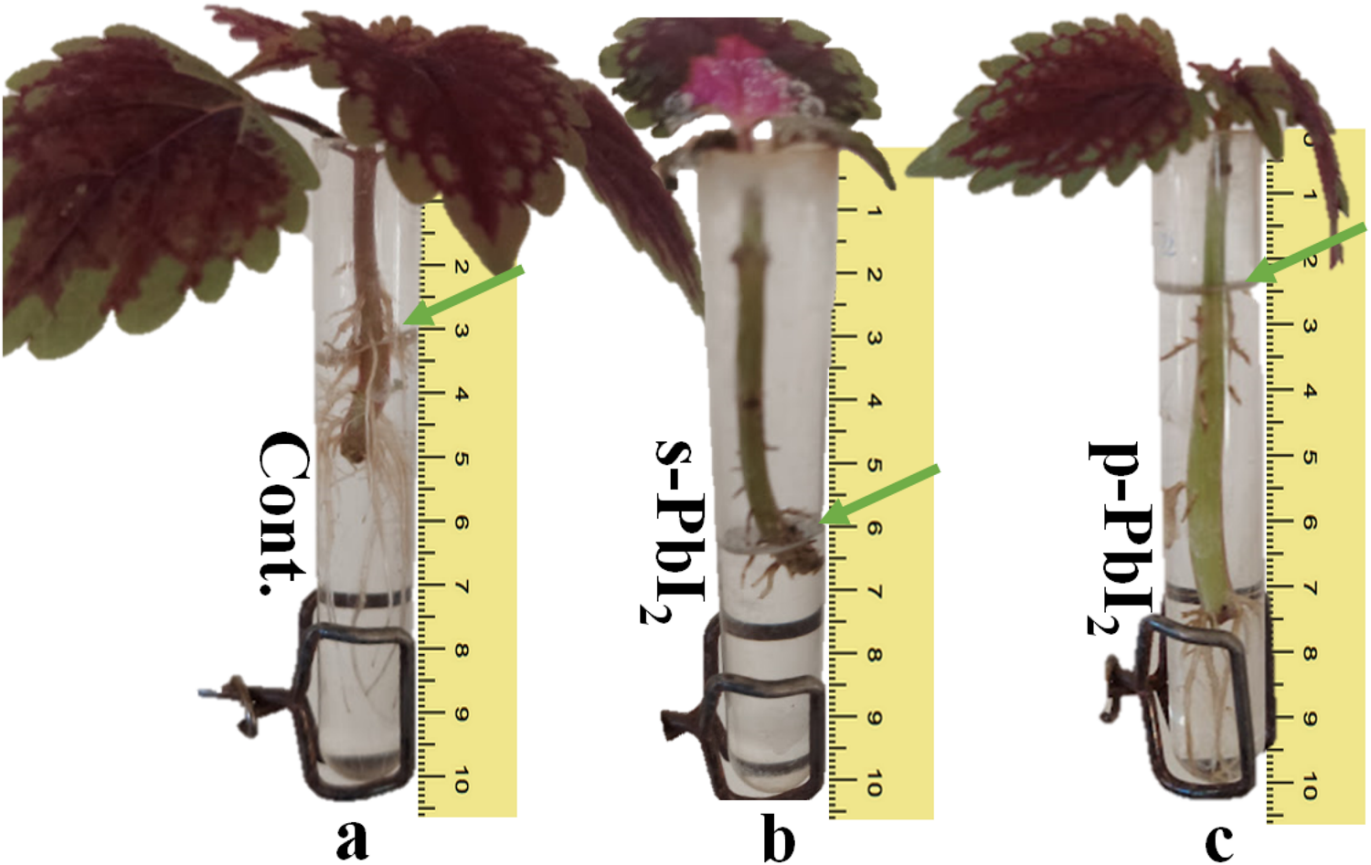
Schematic of water consumption Coleus plant in **Cont.**, **s-PbI**_**2**_**,** and **p-PbI**_**2**_series samples (green arrow).

Remarkably, we obviously found that there is a direct correlation between the amount of absorbed water by roots when were taken in the different sources of Pb involves **p-PbI**_**2**_ and **s-PbI**_**2**_ as well the rate of destroying of plants. The value of height of absorbed water by the plant in the tubes containing samples **Cont., s-PbI**_**2**_**, and p-PbI**_**2**_ (Fig. 5a–c) were decreased from initial value from 10 cm to 7, 4 and 7.6 cm, respectively due to consume of water by Pb, which means that the value of absorbed water controlled by the type of Pb. Finally, **s-PbI**_**2**_ wasted more water than perovskite and reference sample which destroyed perovskite (sample **p-PbI**_**2**_) showed the lowest water absorption, nearing sample **Cont**. To explain these observations, based on the results obtained by R. Riyazuddin et al., more absorption water with the plants such as stomata, xylem vessels, parenchymatous, and mesophyll cells, more toxicity of water containing heavy metals^29^.

### Lead bioavailability

**Figure 6:**
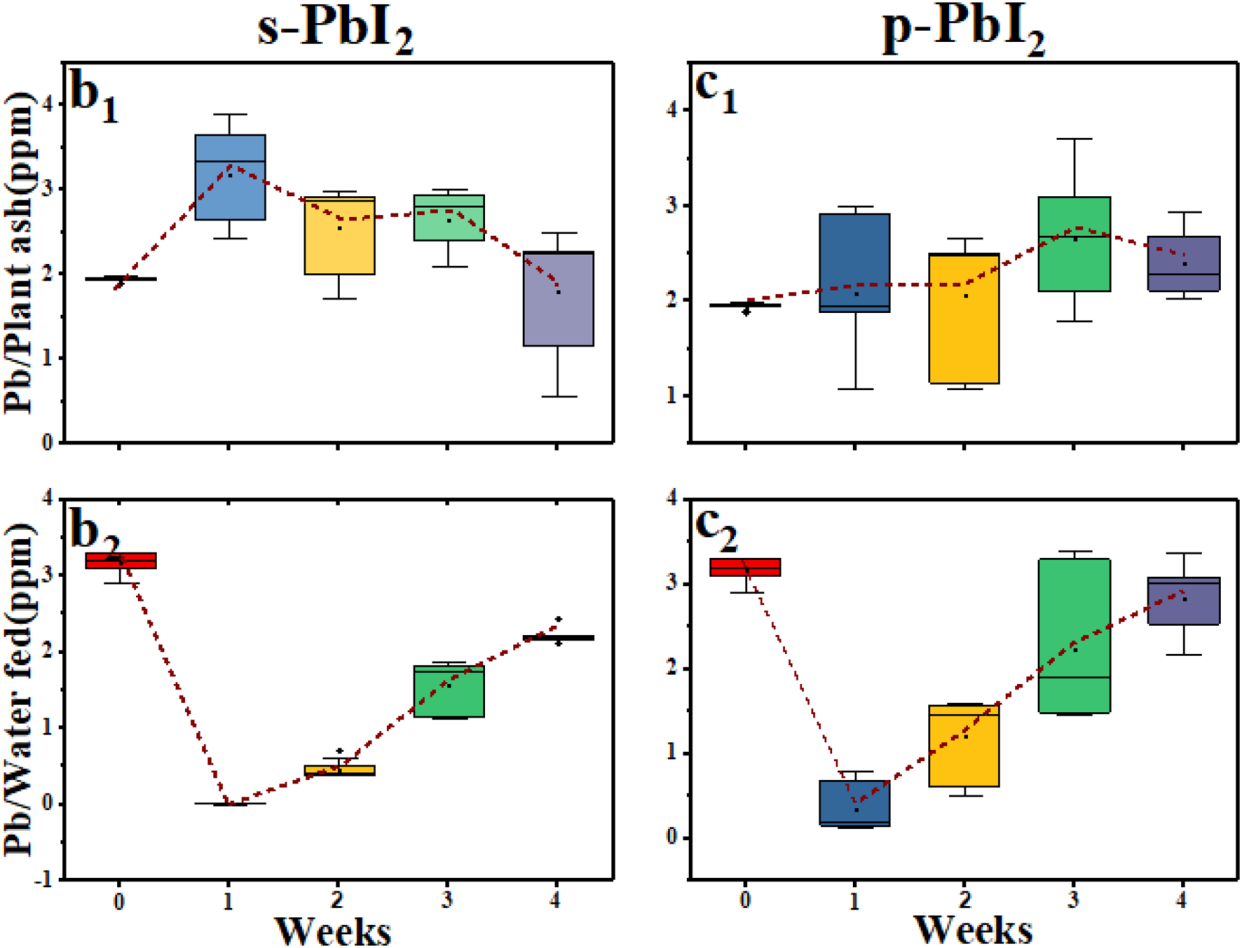
Box plot chart of the amount of lead in (**b**_**1**_) Series **s-PbI**_**2**_ ash (ppm), (**c**_**1**_) Series **p-PbI**_**2**_ plant ash (ppm), (**b**_**2**_) Series **s-PbI**_**2**_ water (ppm), (**c**_**2**_) Water Series **p-PbI**_**2**_ (ppm).

Screening the amount of lead in plants,s ashes of series of **Cont.** and **s-PbI**_**2**_ by using **AAS** as well its bioavailability to evaluate environmental effects have been carried out and the results are shown in Fig. 6b_1_, c_1_, b_2_, and c_2_, respectively. In this study, all three samples of **Cont.**, **s-PbI**_**2**_**,** and **p-PbI**_**2**_ thoroughly were analyzed and each one were repeated three times for more accuracy. In summary, at the end of the first week, as shown in Fig. 6b1, samples of series **s-PbI**_**2**_ shown a significant enhancement in the amount of lead compared to sample series **p-PbI**_**2**_, as shown in Fig. 6c1. In contrast to first week, from the second week to the end of the fourth week (please see Fig. 6b1), a decrease in the amount of lead of **p-PbI**_**2**_ was observed, while the amount of lead of **s-PbI**_**2**_ gradually increases in the same period, as shown in Fig. 6c1. Quantitatively, the amount of lead of series **s-PbI**_**2**_ from first to fourth week was calculated 3.2, 2.6, 2.7, and 1.8 ppm, which for series **p-PbI**_**2**_ in the same period was estimated 2.1, 2.2, 2.7 and 2.4 ppm, respectively.

One of the important conditions which should check is the amount of lead in the water for series **s-PbI**_**2**_, and **p-PbI**_**2**_. In brief, the amount of lead in the water for series **s-PbI**_**2**_ (Fig. 6b2) at the end of the first week, is almost zero which is much less than series **p-PbI**_**2**_**,** as shown in Fig. 6c2, which means that all mass of lead in 5b_2_ is absorbed by the plant, confirming the highest value of 3.2 ppm for series **s-PbI**_**2**_. In following, when the irrigation was increased the amount of lead at the end of the second, third, and fourth week obtained 0.5, 1.5, and 2.5 (ppm), respectively for series **s-PbI**_**2**_ (in Fig. 6b2), and 0.4, 1.2, 2.1, and 3 ppm for series **p-PbI**_**2**_ as shown in Fig. 6c2.

A survey of the amount of Pb has been suspended in water shown that the initial increase of lead in series **s-PbI**_**2**_ is much significant than in series **p-PbI**_**2**_. In summary, more lead absorption in the first week for series **s-PbI**_**2**_ causes the Coleus plant to be poisoned and dried earlier than series **p-PbI**_**2**_, which has a gradual lead absorption.

It is important to point out that a large number studies have been focused on the impact of lead metal on the environmental issue, however the estimation of amount of iodine are not fully understood. As an instant, Eline M. et al. studied the influence of decomposition of MAPbI_3_ as well a variety of perovskite precursors (PbI_2_, Pb(NO_3_)_2_, PbBr_2_, MABr, MAI) on Arabidopsis thaliana which estimated the amount of iodine ion of 79 μM is higher than the criteria value of 50 μM, resulting the impact of iodine species into the environment issues after leakage of perovskite solar cell panels”^31^.

## Conclusion

Here, we investigated the effect of perovskite decomposition from solar cells on Coleus plants, which showed that the effect of decomposed perovskite (**p-PbI**_**2**_) on plant growth was lower than that of commercial PbI_2_ (**s-PbI**_**2**_). We found that the impact of **s-PbI**_**2**_ on the root and leaf of the Coleus plant is more destroyer than **p-PbI**_**2**_. In particular, the amount of water absorption and Pb metal by roots in the **s-PbI**_**2**_ sample is about two-fold more than **p-PbI**_**2**_ ones. Particularly, it is proposed that the toxicity of a contaminated plant with p-PbI_2_ could be found from the converting its roots to transparent thin rhizomes after one week, differently from s-pbI_2_,s rhizomes, showing solid dark features. This interesting results show that the contamination of plants by s-pbI_2_ and p-pbI_2_ will be distinguished from roots and leafs without any analytical characterizations. This new achievement opens new ways and challenges for investigating the effect of perovskite materials of perovskite solar cells on the environmental issue and shedding light on the growing of the plants under the presence of destroyed PSC. More research on the influence of decomposed perovskite on plants, and animals in vivo should be done in the future to get more insights and possibilities about the commercialization of perovskite solar cells and other optoelectronic materials based on superior perovskite materials.

## Data Availability

The datasets used and analyzed during the current study available from the corresponding author on reasonable request.
